# Antioxidant and potential anti-inflammatory activity of extracts and formulations of white tea, rose, and witch hazel on primary human dermal fibroblast cells

**DOI:** 10.1186/1476-9255-8-27

**Published:** 2011-10-13

**Authors:** Tamsyn SA Thring, Pauline Hili, Declan P Naughton

**Affiliations:** 1 School of Life Sciences, Kingston University, London, KT1 2EE, UK; 2 Neal's Yard Remedies, 15 Neal's Yard, London, WC2H 9DP, UK

## Abstract

**Background:**

Numerous reports have identified therapeutic roles for plants and their extracts and constituents. The aim of this study was to assess the efficacies of three plant extracts for their potential antioxidant and anti-inflammatory activity in primary human skin fibroblasts.

**Methods:**

Aqueous extracts and formulations of white tea, witch hazel and rose were subjected to assays to measure anti-collagenase, anti-elastase, trolox equivalent and catalase activities. Skin fibroblast cells were employed to determine the effect of each extract/formulation on IL-8 release induced by the addition of hydrogen peroxide. Microscopic examination along with Neutral Red viability testing was employed to ascertain the effects of hydrogen peroxide directly on cell viability.

**Results:**

Considerable anti-collagenase, anti-elastase, and antioxidant activities were measured for all extracts apart from the witch hazel distillate which showed no activity in the collagenase assay or in the trolox equivalence assay. All of the extracts and products tested elicited a significant decrease in the amount of IL-8 produced by fibroblast cells compared to the control (p < 0.05). None of the test samples exhibited catalase activity or had a significant effect on the spontaneous secretion of IL-8 in the control cells which was further corroborated with the microscopy results and the Neutral Red viability test.

**Conclusions:**

These data show that the extracts and products tested have a protective effect on fibroblast cells against hydrogen peroxide induced damage. This approach provides a potential method to evaluate the claims made for plant extracts and the products in which these extracts are found.

## Background

As the largest organ in the body, the skin provides a barrier against UV radiation, chemicals, microbes and physical pollutants. Challenges of this nature can contribute to skin ageing and inflammation which is characterised by oxidative damage [1-3]. Multiple studies have revealed that the skin is very sensitive to reactive oxygen species (ROS) [1-5]. Since an increase in the formation of the ROS hydrogen peroxide (H_2_O_2_) has been associated with inflamed and diseased tissues [6], H_2_O_2 _can be used to induce oxidative stress in cells and therefore may provide a method to evaluate antioxidant activity of plant extracts [7].

Many herbal extracts and natural products prevent or reduce oxidative stress in *in vitro *models. Mueller *et al*. screened 30 extracts from a wide range of plant families for their anti-inflammatory activities in macrophage cells [8]. The reported anti-inflammatory mechanisms included reduction of the pro-inflammatory cytokines IL-6 and TNF-α, increasing anti-inflammatory IL-10 secretion, and reduction of cyclooxygenase-2 (COX-2) and nitric oxide synthase expression [8]. Chilli pepper extract was shown to have the strongest anti-inflammatory activity along with allspice, apple, basil, bay leaves, black pepper and liquorice, to name but a few. These extracts all led to a reduction in IL-6 secretion. Dimethyl sulfoxide extracts of chilli pepper prevented TNF-α secretion and enhanced IL-10 production. Pure compounds such as apigenin, capsaicin, chrysin, kaempferol, and quercetin reduced IL-6 secretion [8]. *Carthamus tinctorius *- a plant widely used to treat circulation problems has been found to prevent H_2_O_2 _-induced oxidative stress in osteoblastic cells at low concentrations (2-10 μg/mL) [7]. Green tea and its constituents have also been studied for their potential to protect cells from oxidative damage. Polyphenols in green tea can protect against 1 mM H_2_O_2 _induced damage in bladder cells, with epicatechin gallate (ECG) exhibiting strong protective effects across three bladder cell lines [6].

The herbs white tea (*Camellia sinensis *Kuntze), Rose (*Rosa alternifolia *L.) and witch hazel (*Hamamelis virginiana *L.) were recently reported to have high polyphenolic contents and to exhibit high activities in antioxidant assays, along with potential anti-ageing activity via inhibition of collagenase and elastase [9]. These herbs are often included in skin care products and are usually advertised for their astringent and antioxidant properties. In the scientific literature, white tea and witch hazel are reported for topical treatment of skin disorders. White tea has antiseptic and antioxidant properties while witch hazel has long been used for skin trouble such as acne as an astringent and antiseptic [10]. Rose is mostly added in its essential oil form to products for its fragrance while rose water is a traditional eye lotion [10]. The aims of this study were to further explore the anti-inflammatory activity of these plant extracts, commonly used in the skin care industry, and two formulations containing them, to assess their potential antioxidant and anti-inflammatory activity using primary human skin fibroblasts.

## Methods

### Plant extracts and formulations

White tea (freeze dried powder), rose (tincture), witch hazel (dried aerial herb), white tea eye gel and witch hazel distillate were supplied by Neal's Yard Remedies Ltd, Covent Garden. White tea powder (WT) and witch hazel (WH) were extracted in distilled water (500 mg in 10 mL) and then evaporated to dryness, weighed and re-constituted in water at 10 mg/mL. The rose tincture (RT) was evaporated, weighed and re-constituted in water to 10 mg/mL. The products white tea toning eye gel (WTEG) and witch hazel distillate (WHD) were diluted in distilled water to12.5% for WTEG and 50% for WHD for the assays conducted using cell cultures. Since WTEG is viscous, a dilution step (to 12.5% in media with 1% foetal bovine serum (FBS)) was required to allow the gel to permeate through the 0.2 μm filter sterilization membrane to be used in assays at 6.25, 3.125, 1.562 and 0%. The WHD affected the pH of the media and so was diluted to 50% and then used at 25, 12.5, 6.25 and 0% along with 1% FBS. All test extracts were filter sterilized in a cell culture hood prior to being further diluted in media. All extracts were stored at -20°C.

### Collagenase, elastase and antioxidant assays

These assays were performed as described previously [9,11]. Briefly, anti-collagenase activity was studied using a spectrophotometric assay using bacterial collagenase incubated with extracts for 15 minutes. Addition of the synthetic substrate N-[3-(2-Furyl)acryloyl]-Leu-Gly-Pro-Ala (FALGPA) started the reaction and was measured at 335 nm. Similarly, elastase (porcine) was incubated with extracts, then substrate N-Succinyl-Ala-Ala-Ala-p-nitroanilide (AAAPVN) was added and the reaction measured between 381 and 402 nm. Trolox equivalent antioxidant capacity was measured using the 2,2'-azino-bis(3-ethylbenzothiazoline-6-sulfonic acid diammonium salt) (ABTS^+^) free radical assay. A solution of ABTS^+ ^was added to 10 μL amounts of extracts (some of which had to be diluted considerably) and measured at 730 nm. Equivalence was calculated from a trolox standard curve. Catalase activity was assessed using manometry with catalase acting as a positive control [11].

Results for the WTEG and WHD are shown along with the data for WT, RT and WH which were reported previously [9]. In contrast to the previous results, where dried extracts were employed, the formulation WTEG was dissolved in water and of necessity results are shown as concentrations of 1% final volume. WHD was used neat corresponding to 10% final volume in assays.

### Cell culture

The fibroblast cells used in this study were established from an anonymised normal skin sample and were a gift from the Institute of Cancer Research to Dr J. Peacock. Cells were cultured in Dulbecco's Modified Eagle Medium (DMEM) containing 10% FBS (PAA Gold), 5% penicillin-streptomycin solution (Fisher Scientific), and 5% L-glutamine and 5% non-essential amino acids (Sigma Aldrich). To dilute the extracts and for assays, the same medium was used except that FBS was reduced to 1% so as not to interfere with assays. Cells were kept under standard culture conditions at 37°C and 5% CO_2_.

### IL-8 inhibition assay

Cells were seeded at approximately 1 × 10^4 ^cells/mL in 96 well culture plates in standard growth medium and grown until confluent. Once confluent, media was aspirated and 100, 50, 25 and 0 μg of extracts WT, RT, and WH extracts were added (diluted in media). WTEG and WHG formulations were diluted to 12.5% and 50% as described above. Hydrogen peroxide (filter sterilized and diluted in 1% FBS media) was added to bring the concentration within wells to 0.5 mM based on previous reports [6,12,13]. Control wells without H_2_O_2 _were run alongside to assess spontaneous secretion of IL-8 by cells with and without extracts. Plates were incubated for 24 hours and the supernatant collected and stored immediately at -20°C. An ELISA kit (Hycult) was then used to determine the amounts of IL-8 generated by the cells under challenge with H_2_O_2_. Standard curves containing IL-8 were also run according to the supplier's recommendation. Following kit procedure, plate absorbance values were read at 450 nm on a Cary 50 MPR and standard curves were plotted using Graphpad Prism version 5 curve fitting software.

### Cytotoxicity testing

In order to ensure the extracts were not toxic to these particular cells, cytotoxicity testing was performed according to Annan and Houghton with minor modification [12]. Cells were diluted to approximately 1 × 10^4 ^cells/mL in media and 100 μL were added to 96 well culture plates (Nunc) to yield 1 × 10^3 ^cells/well. Cells were left overnight to adhere then the media was aspirated and replaced with media containing 1% FBS, plant extracts at concentrations between 200 and 6.25 μg amounts or products between 25 and 0.39% along with media controls. Cells were grown for 5 days before viability was assessed using the Neutral Red (NR, Sigma Aldrich) assay [14]. Here the media was aspirated and 50 μL filtered neutral red were added (0.15% in PBS pH 5.5) and left to incubate for an hour. The dye was then removed and rinsed with PBS (pH 6.5) three times. The plates were then tapped vigorously to remove PBS prior to adding 100 μL 10% ethanolic PBS (pH 4.2) which dissolves the NR dye for uptake by the cells. After 30-60 seconds of gentle tapping and shaking, the absorbance was read at 510 nm on a Cary 50 MPR.

### Microscopy to assess antioxidant activity

Cells (approximately 1 × 10^4 ^cells/mL) were seeded onto sterile, glass coverslips placed in 6-well culture plates and left to adhere overnight. The following day, wells were treated with 1% FBS media, 100 μg of WT, RT or WH or 6.25% WTEG or WHD both with and without 0.5 mM H_2_O_2 _and incubated for 24 hours. After incubation, wells were emptied and IMS added to fix the cells for 5 minutes. PBS was then used to rinse the cells twice before treating with Giemsa stain (Sigma Aldrich-diluted 1/6 in PBS) for 30 minutes. Coverslips were washed twice in PBS and left to dry. Once dry, histomount was used to mount the coverslips to the slides. Cells were then examined and photographed using a Nikon Eclipse 80i microscope.

### Statistical analysis

Statistical analysis was performed using SPSS (version 17) software. Significance was taken at the 0.05 level. Levels of IL-8 between control and test samples were analysed using ANOVA and post hoc LSD. The differences between samples with and without H_2_O_2 _to show spontaneous secretion of IL-8 were analysed using the Mann-Whitney test.

## Results and Discussion

### Enzyme inhibition and antioxidant capacity

In comparison to the previous results for the plant extracts WH, WT and RT, the WTEG retained activity in all three assays, inhibiting collagenase and elastase by over 10% when diluted to 1%, along with a TEAC score of > 5 μmoles (Table 1). In contrast, WHD at 10% exhibited a high anti-elastase activity of ca. 41% with no detectable anti-collagenase or TEAC activities under the conditions used. The WHD does however contain 14-15% ethanol, although the final concentration of ethanol in the assay is very low (1.4-1.5%) accounting for some 10% of the elastase inhibition observed [15]. All of the herb extracts, formulations or media that were used were assessed for catalase activity with no activity being detected. Thus, inactivation of the H_2_O_2 _by catalase activity by test samples in the cell assays can be ruled out.

**Table 1:** Summary of results from collagenase, elastase, and antioxidant capacity (TEAC) (N = 6, ± SEM).

Extract	Concentration added	% inhibition of elastase	% inhibition of collagenase	Trolox equivalent (μmoles)
WTEG	1%	10.57 ± 0.45	10.96 ± 4.82	5.78 ± 0.55

WHD	10%	41.00 ± 2.38	No activity	No activity

WT^†^	25 μg	89.00 ± 3.62	87.08 ± 4.79	10.62* ± 0.69

RT^†^	25 μg	22.08 ± 0.64	40.96 ± 3.9	9.91** ± 0.85

WH^†^	25 μg	2.8 ± 0.77	13.70 ± 3.26	13.15** ± 0.48

* denotes extracts diluted to 1 μg and ** 6.25 μg: ^† ^from ref [9]

### IL-8 inhibition

**Figure 1:**
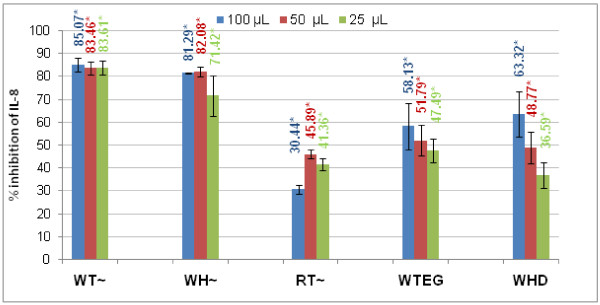
**Inhibition of IL-8 release by the WT, RT, WH, WTEG and WHW**. Comparisons are against control values for (N = 6 ± SEM, *P < 0.05 compared with H_2_O_2 _control). Exact % values are given for clarity. Extracts (~) were tested at 100, 50 and 25 μg final volume, WTEG was tested at 6.25, 3.125 and 1.256% and WHD at 25, 12.5, and 6.25% respectively. Legend indicates volume added in assay to yield afore-mentioned concentrations.

Upon addition of H_2_O_2 _(0.5 mM) a significant decrease in IL-8 production was observed for all preparations compared to the media controls (p = 0.000) (Figure 1). The WT and the WH extracts exhibited the best activity ranging from 85-83% inhibition of IL-8 production. The RT extract showed the lowest inhibition which was in the range of 30-45%. For the WTEG and WHD products, the inhibition levels range from 47-58% and 36-63% respectively over the concentration range studied. Low concentrations of WTEG and WHD due to the necessary dilution steps used for the WTEG and WHD resulted in activity of *circa *50%. An increase in activity might have been achieved had higher concentrations been possible. For WTEG and WHD a potential trend towards a dose response was noted but statistical analysis revealed this was not significant (p > 0.05).

**Figure 2:**
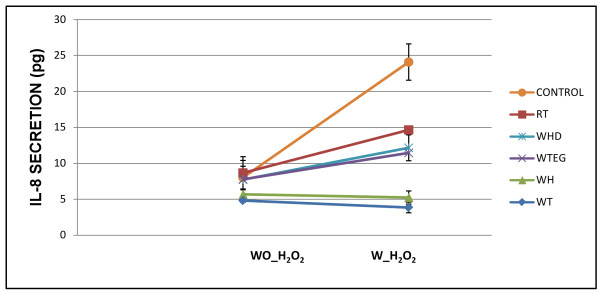
**Effect of treatment on IL-8 production without (wo_) and with (w_) H_2_O_2_**.

A substantial amount of IL-8 is released during the experiment owing to experimental conditions. Addition of the extracts and formulations had no significant effect on this baseline IL-8 release (Figure 2) over the concentration range studied. In comparison, IL-8 release was greatly enhanced after addition of H_2_O_2 _to control samples. All test samples significantly reduced levels of IL-8 release upon addition of H_2_O_2_. There was no significant difference between the spontaneous secretion of IL-8 in the media control cells compared to the test controls (p > 0.05), which indicate low cell stress levels. Perhaps a reason for this is that in low FBS media, the cells utilise components of the extracts added. No significant differences were found between the pre- and post-H_2_O_2 _levels of IL-8 release for all test samples (p > 0.05) showing high levels of protection in each case.

**Figure 3:**
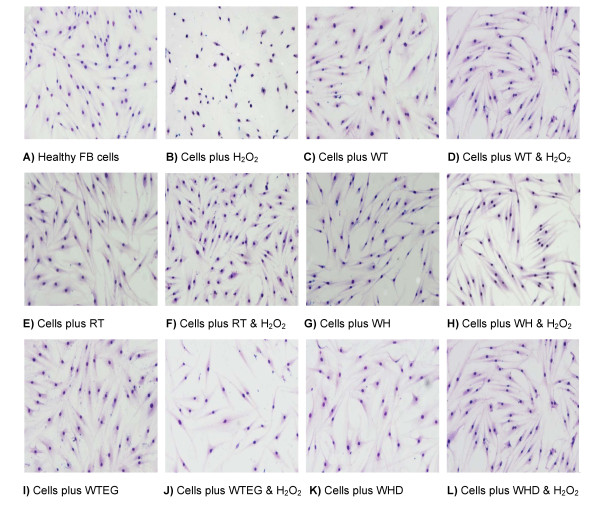
**Light microscope images of fibroblast cells (×400) with the extract and product treatments**. (**A**-**H**) cells treated with 100 μg of plant extracts with and without 0.5 mM H_2_O_2_. (**I**-**L**) cells treated with 100 μg of plant extracts with and without 0.5 mM H_2_O_2_.

Cells were examined microscopically to assess the protective effects of each test sample against H_2_O_2 _induced oxidative stress (Figure 3). There is a visible difference in the control cells upon addition of H_2_O_2_, with loss of typical fibroblast appearance, the nuclei becoming condensed and staining dark blue, revealing the occurrence of apoptosis. In contrast, upon treatment with test samples, the cells appear to remain intact and retain their shape on exposure to H_2_O_2 _(Figure 3, image 2). The WT and WH treatments exhibit greater activity against H_2_O_2 _induced damage than the products WHW and WTEG (Figure 3).

The addition of plant extracts to cell media can produce oxidative stress indirectly and has been shown by Lapidot *et al*. [16], where the phenolics interact with ingredients in the media producing H_2_O_2_. However, microscopic examination revealed that the H_2_O_2_-treated cells resemble the controls indicating that any oxidative stress caused by extracts and media may be present but was not detrimental to the cell structure (Figure 3).

Cytotoxicity profiles for each test sample were assayed using the Neutral Red dye assay (Table 2). No significant cytotoxic effects were observed. No toxicity was observed for the products WTEG and WHD. For the extracts, an increase in concentration of WT resulted in increased viability indicating potential nutritive use by the cells which negates supposed low media levels.

**Table 2:** Cytotoxicity studies showing the % cell viability (N = 6, ± SEM) when treated with various concentrations of extracts and products using the neutral red assay.

Product	25%	12.50%	6.25%	3.12%	1.56%	0.78%	0.39%
WTEG	^1^NT	^1^NT	93.84 ± 2.05	92.99 ± 2.24	98.87 ± 1.81	98.27 ± 2.89	100.66 ± 2.83

WHD	99.77 ± 0.83	99.88 ± 2.53	102.35 ± 1.21	101.47 ± 3.36	102.10 ± 3.41	^1^NT	^1^NT

**Extract**	**200 μg**	**100 μg**	**50 μg**	**25 μg**	**12.5 μg**		

WT	112.97 ± 1.55	102.39 ± 1.59	88.08 ± 2.03	83.076 ± 6.26	76.37 ± 5.63		

RT	93.17 ± 4.65	96.67 ± 0.92	94.24 ± 5.77	92.52 ± 6.27	94.41 ± 7.78		

WH	96.90 ± 2.055	95.57 ± 3.95	91.47 ± 5.77	101.42 ± 3.65	99.26 ± 2.83		

^1^NT indicates not tested at these concentrations due to solubility ability

The observed anti-IL-8 and antioxidant activity may be owing to the polyphenolic compounds within the extracts. In general, flavonoids from plants have been found to demonstrate antioxidant activity such as kaempferol and quercetin glycosides from *Carthamus tinctorius *which showed a reduction in oxidative stress and damage in osteoblastic cells [7]. Fruit extracts from *Momordica charantia *L. have shown *in vitro *antioxidant activity against H_2_O_2 _induced damage in fibroblasts and keratinocytes [13]. As mentioned previously many plant extracts (30 screened in total) including chilli pepper, basil and licorice as well as individual flavonoids such as apigenin, kaempferol and quercetin have led to a reduction in IL-6 release by macrophages. Many of these 30 plant extracts also exhibited a positive effect reducing TNF-α and COX-2 levels as well as increasing IL-10 secretion [8].

Flavonoids are thought to prevent the formation of ROS by inhibiting enzymes or chelating trace metals which can mediate free radical production as well as being free radical scavengers and upregulating genetic antioxidant defences [17]. Tea contains both kaempferol and quercetin as well as flavanols or catechins up to 30% dry weight and other acids such as gallic acid, caffeic acid and coumaric acid [10] which may account for the high activity seen in this study especially as a freeze-dried white tea powder was used. Catechins are powerful bioflavonoids and green tea has been shown *in vivo *and *in vitro *to have anti-inflammatory and antioxidant activity [18] (cited by Coyle *et al*. [6]). Epigallo-catechin-3-gallate (EGCG) is a catechin found in green and white tea and has been reported to inhibit IL-8 gene expression in respiratory epithelium cells [19]. EGCG and other catechins can also inhibit proteins involved in inflammation, including TNF-α and xanthine oxidase [19].

Witch hazel leaves and bark contain up to 10% tannins, which contribute to its astringent properties. Bark contains mostly hamamelitannins and catechols while the leaves have more proanthocyanidins, ellagitannins and some essential oils [15]. Witch hazel has been used topically as an astringent and anti-bacterial treatment for skin to alleviate inflammation caused by acne and eczema. Hamamelitannin has been investigated and found to have a significant effect on superoxide anion radical induced damage in murine fibroblast cells and exhibited higher than the activity of gallic acid, used as a positive control [20]. The witch hazel used in this study consisted of leaves and small twigs which suggest a lower amount of hamamelitannin. However, other tannins and polyphenols such as gallic acid may be responsible for the effects as they are strong antioxidants [9].

*Rosa centifolia *flowers are the primary plant constituents in the tincture supplied for this study and contain tannins, anthocyanins, and essential oils [18]. A flower extract from *Rosa hybrida *was found to have significant anti-inflammatory and analgesic activity [21].

The WT eye gel used in this study is made up of several ingredients including aloe vera, eyebright, rose, and witch hazel extracts as well as sodium hyaluronate which may all contribute towards its activity. No antioxidant activity was observed for hyaluronate which is the major component of glycosaminoglycans which are responsible for the hydration of the skin and also in wound healing and scar formation meaning it stimulates fibroblast proliferation [22]. All these factors may combine synergistically to demonstrate the effects seen in this study. For the witch hazel distillate, with only addition of water and alcohol, infer the activity arises from the WH. It is also important to note that the observed anti-inflammatory effects are still significant and justify the purpose for which they are advertised.

## Conclusion

These data show that the extracts and products tested have a protective effect on fibroblast cells against H_2_O_2 _induced damage. This approach provides a potential method to evaluate the claims made for plant extracts and the formulations in which these extracts are found.

## Competing interests

PH is an employee of Neal's Yard Remedies Ltd, which in part funded the studentship for TT.

The authors declare that they have no competing interests.

## Authors' contributions

TT, PH, and DPN participated in the design of the study data analyses and manuscript preparation. TT conducted the assays and analyses. All authors read and approved the final manuscript.
